# Health equity: access to quality services and caring for underserved populations

**DOI:** 10.1093/heapol/czad073

**Published:** 2023-11-16

**Authors:** Maria Lazo-Porras, Tricia Penniecook

**Affiliations:** CRONICAS Centre of Excellence in Chronic Diseases, Universidad Peruana Cayetano Heredia, Av. Armendariz 445, Lima 15074, Perú; College of Public Health, University of South Florida, 13201 Bruce B. Downs Blvd, MDC 56, Tampa, FL 33612, United States

**Keywords:** Health equity, health systems research, vulnerable populations

## Abstract

Barriers to access to quality services and caring for underserved populations are a call to action for researchers and other key partners to achieve health equity. In order to accomplish this, several key partners play important roles. More participation of younger generations, women and people of color from different contexts should be encouraged and facilitated. This editorial serves to present this journal issue that includes the articles of young women from low- and middle-income countries. Different methodologies are used to demonstrate the problem of access to quality services and care in a comprehensive way. After understanding the public health problems using an equity lens, we need to implement evidence-based interventions to improve the health system response.

Access to health and quality care is a basic right for people worldwide. However, in reality, within and between communities, countries and regions there is a wide range of inequalities in terms of what each person receives (Baeten *et al.*, 2018; Houghton *et al.*, 2020). Additionally, some populations have more limited access to health care—the underserved populations, especially people who live in low- and middle-income countries (LMICs), and minority and vulnerable groups within countries (Heslehurst *et al.*, 2018; Green *et al.*, 2021; Khanijahani *et al.*, 2021).

Access to health care is a complex concept that encompasses multiple aspects, and adding access to quality care creates another level of complexity (Kruk *et al.*, 2018). Factors include not just availability of health services and adequate service delivery, but also barriers to the utilization of services, such as financial, organizational, social or cultural issues. Physical accessibility and acceptability of services are also obstacles as well as the effectiveness of care to achieve satisfactory health outcomes (Gulliford *et al.*, 2002).

Some examples of limitations to health-care access are shared in this issue—studies that show the inadequate implementation of health policies for mental health, maternal and child care, and accountability services. These are examples of how health-care policies fall short and they highlight the need for action in order to deliver a comprehensive health service to the population (Chilumpha *et al.*, 2023; Mayo *et al.*, 2023; Okoth *et al.*, 2023). Other studies expose how service delivery has not been designed to be people-centred and services do not adequately cover the population’s requirements (Chicmana-Zapata *et al.*, 2023; Okoth *et al*., 2023). Other papers show that less educated or under-resourced groups have limited access to services or are left behind (Adaobi Ogbozor *et al.*, 2023; Renu *et al.*, 2023). Additionally, two articles describe how the COVID-19 pandemic had a greater impact on minority groups and high-risk populations compared to other groups. One study focuses on the implementation of policies to provide mental health services, finding that the policy exists on paper but not in practice (Mayo *et al.*, 2023); whereas the other study explores a government’s response to the health and food needs of indigenous populations during the COVID-19 pandemic. It showed that the government did not use a justice lens when designing and implementing its response to the health crisis (Chicmana-Zapata *et al.*, 2023).

Another interesting aspect to highlight in this issue is the diversity of methods used to study access to health care. Two studies included policy analysis or document review (Chicmana-Zapata *et al.*, 2023; Mayo *et al.*, 2023), five studies used qualitative methods with interviews (Adaobi Ogbozor *et al.*, 2023; Chicmana-Zapata *et al.*, 2023; Chilumpha *et al.*, 2023; Mayo *et al.*, 2023; Okoth *et al.*, 2023), and two studies used focus groups and observations (Adaobi Ogbozor *et al.*, 2023; Chilumpha *et al.*, 2023). Another study used quantitative methods to identify the risk factors for not using the emergency contraceptive pill (Renu *et al.*, 2023). The qualitative studies involved a wide diversity of key partners trying to understand the problem from different angles. Adaobi Ogbozor *et al*. describe the different perspectives on informal payments in maternal care, contrasting the views of users, health-care workers, managers and policy-makers in an interesting way (Adaobi Ogbozor *et al.*, 2023).

Barriers to accessing quality services and care in underserved populations represent a call to action for researchers and other key partners to achieve health equity. Health equity is described as ‘*a fundamental component of social justice that indicates the absence of avoidable, unfair or remediable differences among groups of people due to their social, economic, demographic or geographic circumstances*’ (PAHO/WHO, 2023). Various key partners play an important role in achieving this goal. However, in global health the voices of groups that have not traditionally been perceived or supported in research careers are needed, such as the younger generation, women and people of colour from different contexts to reveal the nuanced barriers to achieving health equity (Javadi and Hussain, 2020; Amisi *et al.*, 2023). Promoting participation by more women from LMICs is a crucial step towards increasing the number and quality of voices of those who are close to or part of the most vulnerable groups (Kwamie and Jalaghonia, 2020). After arriving at a comprehensive understanding of the problem, evidence-based interventions will be needed to improve the health system response (Peterson *et al.*, 2023). This will require participation by different stakeholders (including communities), and multi-sector actions and methods to design and evaluate how to close the health equity gap.

## Data Availability

This editorial does not draw on any primary or secondary data.
